# Chromosomal microarray analysis in developmental delay and intellectual disability with comorbid conditions

**DOI:** 10.1186/s12920-018-0368-4

**Published:** 2018-05-24

**Authors:** Yanjie Fan, Yanming Wu, Lili Wang, Yu Wang, Zhuwen Gong, Wenjuan Qiu, Jingmin Wang, Huiwen Zhang, Xing Ji, Jun Ye, Lianshu Han, Xingming Jin, Yongnian Shen, Fei Li, Bing Xiao, Lili Liang, Xia Zhang, Xiaomin Liu, Xuefan Gu, Yongguo Yu

**Affiliations:** 1 Department of Pediatric Endocrinology/Genetics, Xinhua Hospital affiliated to Shanghai Jiao Tong University School of Medicine, Shanghai Institute for Pediatric Research, 1665 Kongjiang Road, Shanghai, 200092 China; 2 Department of Pediatrics, People’s Hospital of Shanghai Pudong New District, 490 South Chuanhuan Road, Shanghai, 201200 China; 3 Department of Pediatrics, Peking University First Hospital, 8 Xishiku Dajie Xicheng District, Beijing, 100034 China; 4 Department of Developmental and Behavioral Pediatrics, Shanghai Children’s Medical Center, Shanghai Jiao Tong University School of Medicine, 1678 Dongfang Road, Shanghai, 200127 China; 5 Department of Endocrinology, Shanghai Children’s Medical Center, Shanghai Jiao Tong University School of Medicine, 1678 Dongfang Road, Shanghai, 200127 China; 6 MOE-Shanghai Key Laboratory of Children’s Environmental Health, Xinhua Hospital affiliated to Shanghai Jiao Tong University School of Medicine, 1665 Kongjiang Rd, Shanghai, 200092 China

**Keywords:** Chromosomal microarray, Developmental delay, Intellectual disability, Pathogenic copy number variations, Comorbid conditions

## Abstract

**Background:**

Developmental delay (DD) and intellectual disability (ID) are frequently associated with a broad spectrum of additional phenotypes. Chromosomal microarray analysis (CMA) has been recommended as a first-tier test for DD/ID in general, whereas the diagnostic yield differs significantly among DD/ID patients with different comorbid conditions.

**Methods:**

To investigate the genotype-phenotype correlation, we examined the characteristics of identified pathogenic copy number variations (pCNVs) and compared the diagnostic yields among patient subgroups with different co-occurring conditions.

**Results:**

This study is a retrospective review of CMA results generated from a mixed cohort of 710 Chinese patients with DD/ID. A total of 247 pCNVs were identified in 201 patients (28%). A large portion of these pCNVs were copy number losses, and the size of copy number losses was generally smaller than gains. The diagnostic yields were significantly higher in subgroups with co-occurring congenital heart defects (55%), facial dysmorphism (39%), microcephaly (34%) or hypotonia (35%), whereas co-occurring conditions of skeletal malformation (26%), brain malformation (24%) or epilepsy (24%) did not alter the yield. In addition, the diagnostic yield nominally correlated with ID severity.

**Conclusion:**

Varied yields exist in DD/ID patients with different phenotypic presentation. The presence of comorbid conditions can be among factors to consider when planning CMA.

**Electronic supplementary material:**

The online version of this article (10.1186/s12920-018-0368-4) contains supplementary material, which is available to authorized users.

## Background

Developmental delay (DD) and intellectual disability (ID) are estimated to affect ~ 1% of the children across the world [1]. Genetic factors play a major part in DD/ID (up to ~ 47.5%) [2]. Identifying the genetic cause is crucial for accurate etiological diagnosis and refined clinical management. Chromosomal microarray analysis (CMA) has been recommended as a first-tier genetic test for unexplained DD/ID and congenital malformations [3, 4]. The reported diagnostic yields of clinical CMA vary between 12 and 20%, depending on the population and methods used [3, 5]. A wide spectrum of phenotypes can be present in the DD/ID cohorts, including different degrees of ID severity [1] and co-occurrence of other conditions, like epilepsy, autism or dysmorphic features [6]. The diagnostic yields in subgroups of patients with different clinical manifestation are not clear yet. Further assessment of the diagnostic yields in DD/ID patients with different ID severity and co-occurring condition is desired, for it could offer clinicians the phenotypic clues of pathogenic copy number variations (CNVs).

In this study, we reviewed CMA results generated from a Chinese cohort of 710 patients with DD/ID as the main manifestation. We characterized the property and physical distribution of pathogenic CNVs (pCNVs), and compared the yield of CMA among patients with different ID severity and comorbid conditions. Together we delineated the genotypes, diagnostic yields and phenotypes in a DD/ID cohort with heterogeneous manifestations.

## Methods

### Patients

This is a retrospective study conducted in Endocrine and Genetic Department, Xinhua Hospital and Shanghai Children’s Medical Center, China. CMA results from 710 patients (432 males and 278 females, age range from 1 month to 29 years old, average 4.2 years old, visited the clinic during the period of March 2011 to February 2016) were reviewed. Two criteria were met for inclusion in this study: 1) individuals who presented DD/ID as the main manifestation, with or without additional features such as congenital heart defects (CHD), autism, dysmorphism et al.; 2) individuals with whole-genome microarray analysis done. The exclusion criteria were individuals who had central nervous system infection, brain injury or intracranial tumor. This study was reviewed and approved by the ethical committee of Xinhua hospital and Shanghai Children’s Medical Center, China, and informed consent was obtained from the patients or parents (for patients under 18 years old).

The phenotypic information of patients were assessed routinely in the clinic, including family history, pre/peri-natal history, physical examination, standardized measure of intelligence/development, instrumental evaluations (brain MRI, EEG, ultrasound etc.). These clinical records were collected, categorized and accessed electronically in this study. Patient were classified based on ID severity: mild (IQ level 55–70), moderate (IQ level 40–55), severe (IQ level 25–40) and profound ID (IQ level below 25), when the standardized measure of intelligence was valid and available. For comparison of the diagnostic yields in Table 1.II/III, only patients with clinical record indicating negative for the specific condition is counted as “without select condition”. For example, in the group of “abnormal blood biochemistry”, only patients who went through tandem mass spectrometry of blood samples with a normal biochemical profile returned were counted as “without select condition”. For the groups of kidney/urinary tract, gastrointestinal or respiratory tract anomalies, clinicians did not perform detailed examination of these systems in most patients, thus we did not conduct statistical analysis in these groups. For “pre−/peri-natal problems”, conditions included intrauterine growth retardation, pre- or post-term birth and low APGAR score etc. “Family history” was considered positive when first- or second-degree relative with DD/ID was reported. For “karyotypical abnormalities”, patients with abnormal results of karyotype were counted as “with select condition”. These patients were included as they comprised a portion of DD/ID patients referred to CMA, with the main purpose to confirm the chromosomal abnormality and further delineate the spanning of gain or loss.

**Table 1:** Diagnostic yields in patients categorized by ID severity and co-occurring conditions

I. Based on ID severity								
	total		P#		Yield		*P*-value	
Mild	140		27		19%		0.08	
Moderate	105		23		22%			
Severe	85		28		33%			
Profound	15		2		13%			
Not categorized	365		121		33%		/	
II. Based on co-occurring conditions present in > 50 patients, with statistical analysis performed								
	With select condition			Without select condition			Odds ratio	*P*-value
	total	P#	Yield	total	P#	Yield		
SS^a^	201	65	32%	136	30	22%	1.69	0.048
CHD^b^	98	54	55%	110	20	18%	**5.52**	**2.66E-08**
Gonadal dysplasia	50	18	36%	103	20	19%	2.33	0.03
Skeletal malformation	62	16	26%	24	3	13%	2.44	0.251
Facial dysmorphism	201	79	39%	194	39	20%	**2.57**	**4.27E-05**
Microcephaly	128	43	34%	140	25	18%	**2.33**	**0.0033**
Brain malformation	147	36	24%	148	29	20%	1.33	0.328
Epilepsy	62	15	24%	115	23	20%	1.28	0.567
Hypotonia	54	19	35%	113	18	16%	**2.87**	**0.00613**
Pre/peri-natal problems	160	50	31%	234	50	21%	1.67	0.034
SS + dysmorphism	79	30	38%	71	9	13%	**4.22**	**6.70E-04**
SS + microcephaly	66	25	38%	74	14	19%	2.61	0.015
SS + brain malformation	56	17	30%	45	9	20%	1.74	0.261
CHD + dysmorphism	50	31	62%	61	8	13%	**10.81**	**9.10E-08**
III. Based on co-occurring conditions present in < 50 patients								
		total	P#	Yield		total	P#	Yield
Family history		38	7	18%	Macrocephaly	14	2	14%
Autism		20	3	15%	Hypertonia	26	6	23%
Muscle weakness		10	4	40%	Cleft lip/palate	8	7	88%
Obesity		16	10	63%	Low weight	47	23	49%
Ocular/auditory anomalies		50	8	16%	Kidney/urinary tract anomalies	12	5	42%
Gastrointestinal anomalies		10	3	30%	Respiratory tract anomalies	6	4	67%
Abnormal blood biochemistry		33	9	27%	Karyotypical abnormalities	26	24	92%

^a^SS-short stature; ^b^CHD-congenital heart defects

*P*# number of patients with pCNVs identified, *Odds ratio* yielding pCNVs in patients with select condition versus without select condition, based on fisher’s exact; *p*-value < 0.01, two-tailed were displayed in bold

### Chromosomal microarray analysis

500 μl peripheral venous blood was withdrawn to ethylenediamine tetra-acetic acid (EDTA) tubes. Genomic DNA was extracted with GentraPuregene Kit (Qiagen, Germany) or Lab-Aid 820 kit (ZSandx, China). Detection of genomic CNVs was performed with AffymetrixCytoScan HD or 750 K arrays (average probe spacing 1.1 kb and 4.1 kb, respectively) following the manufacturer’s instructions. Array results were visualized and analyzed by Chromosome Analysis Suite software (Affymetrix, USA). The parental origin of CNVs was examined by CMA or quantitative real-time polymerase chain reaction.

### Variant filtering

Size threshold for CNV analysis was set at > 100 kb for gains, > 50 kb for losses and > 10 Mb for loss of heterozygosity. Next, analysis was restricted to rare CNVs - those with < 80% overlap of any common CNVs (< 1% frequency) in the DGV (Database of Genomic Variants) or a database of 2691 phenotypically normal controls (offered by Affymetrix). Interpretation and report of CNVs followed the ACMG guideline [7]. The CNVs deemed benign were not reported.

### Statistical analysis

All statistical analyses were performed on Vassarstats (http://www.vassarstats.net/). The correlation between select condition and pCNV finding was analyzed by Fisher’s exact test on a 2 × 3 or 2 × 2 contingency table (row: the number of patients with or without certain condition; column: the number of patients with or without pCNVs identified). Analysis was only done in conditions with patients number above 50. Odds ratio was calculated, and statistical differences were defined as *p* < 0.01, two-tailed test.

## Results

### The clinical overview and overall diagnostic yield

In this retrospective study, a total of 710 patients with DD/ID as the main manifestation were included. Among this cohort, standardized measure of intelligence was available in 345 patients. Based on the degree of ID severity, the patients can be categorized to “mild” (140), “moderate” (105), “severe” (85) and “profound” (15) (Table 1I). Only 161 patients present DD/ID as the main manifestation without other phenotype reported, and the rest of the cohort were with one or more comorbid conditions, as listed in Table 1 part II/III. The most common conditions co-occurring with DD/ID were short stature (201 patients), facial dysmorphism (201 patients), pre/peri-natal problems (160 patients), brain malformation (147), microcephaly (128) and congenital heart defects (98).

**Fig. 1:**
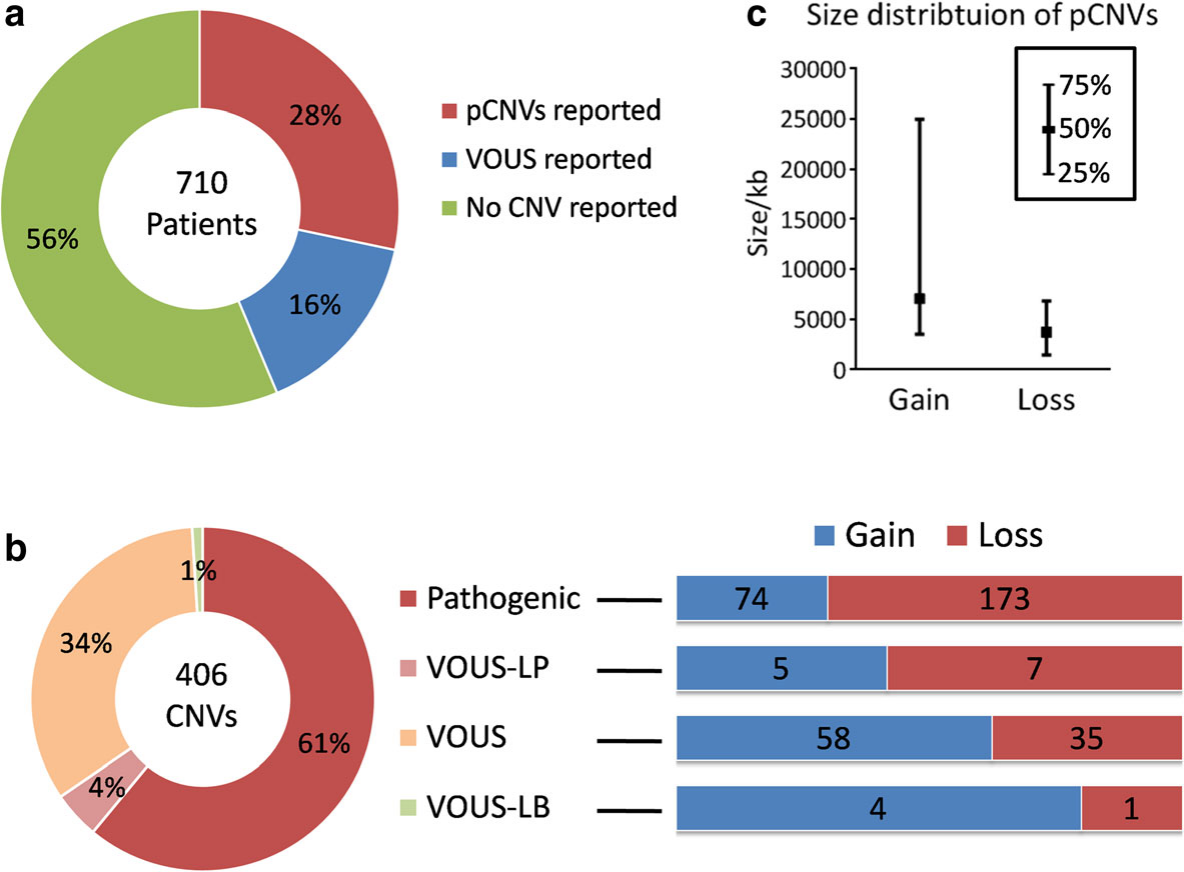
Characterization of CNVs identified in 710 patients with DD/ID. **a** Diagnostic yield of CMA. pCNVs: pathogenic CNVs; VOUS: variants of uncertain significance. **b** Interpretation of 406 rare CNVs identified, and the number of “Gain” and “Loss” in each category. VOUS-LP: variants of uncertain significance, likely pathogenic; VOUS-LB: variants of uncertain significance, likely benign. **c** Size distribution of 247 pCNVs

The overall diagnostic rate was 28% - 201 patients were found to harbor pathogenic CNVs. One hundred nine patients (16%) were found with variants of uncertain clinical significance (VOUS), and 400 patients (56%) received a negative result (no CNV or only benign CNVs found). A total of 406 CNVs were reported to the patients (Fig. 1b), including 247 pathogenic CNVs (pCNVs, 61%), 18 VOUS-likely pathogenic (VOUS-LP, 4%), 136 VOUS (36%) and 5 VOUS-likely benign (VOUS-LB, 1%).

### Characterization of pCNVs

**Fig. 2:**
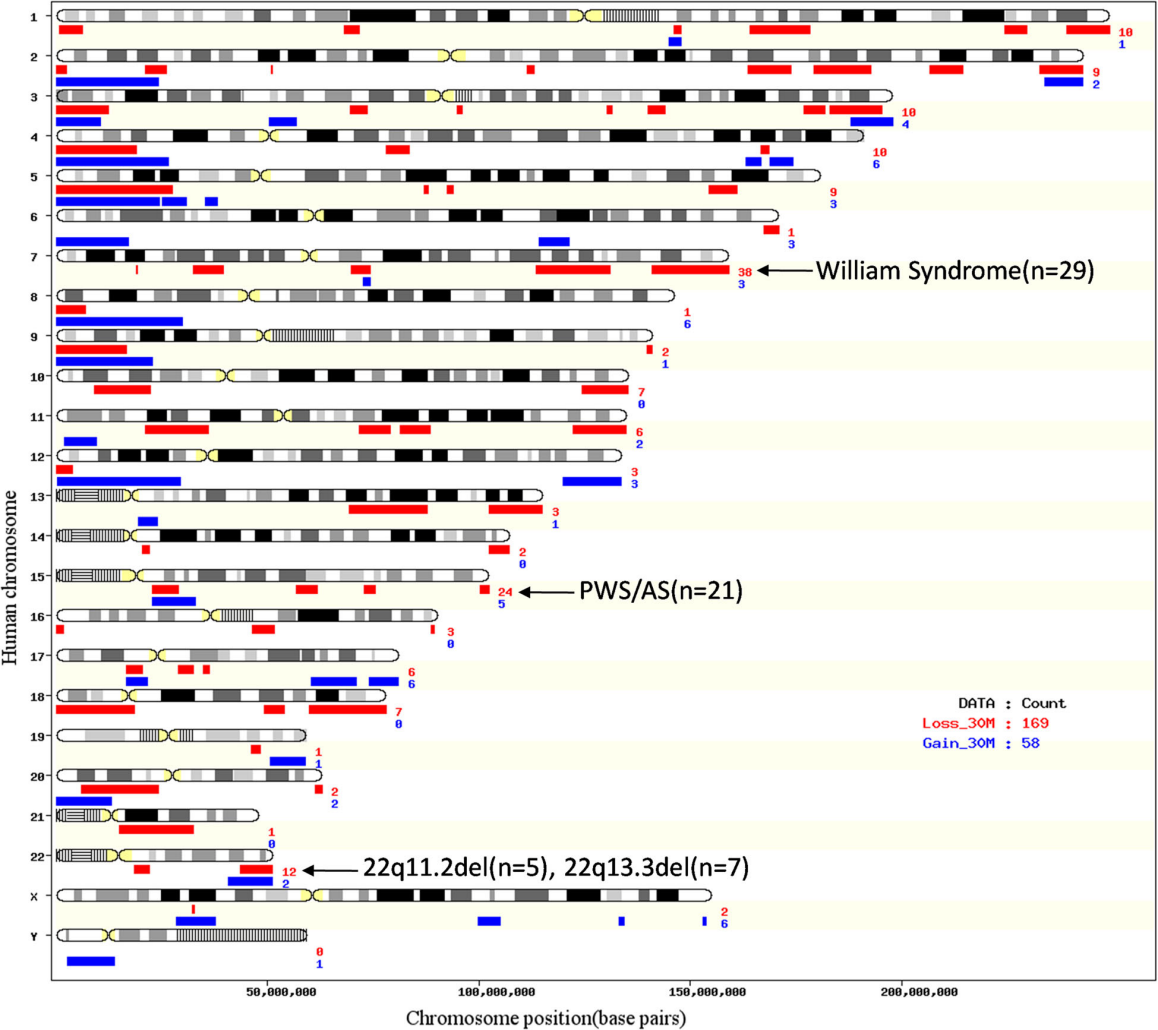
Physical distribution of pCNVs on the chromosomes (only pCNVs with a size smaller than 30 Mb were displayed). Total count of pCNVs on each chromosome was labeled, and the most common pCNVs were located on chr7, 15 and 22. The main syndromes involved in these common pCNVs were annotated

We characterized the property, size and physical distribution of pCNVs. Among 247 pCNVs identified, 173 were losses and 74 were gains (Fig. 1b). A bias towards loss was observed in pCNVs - the proportion of loss increased accordingly with the interpretation of CNV towards pathogenicity, from “VOUS-LB”, “VOUS”, “VOUS-LP” to pathogenic (Fig. 1b). Regarding the size of CNVs, losses were generally smaller than gains (Fig. 1c), with a median size of 3724 kb compared to 7047 kb of gains. Regarding the physical distribution, pCNVs distributed over all chromosomes, and most terminals were covered by gains or losses found in these 201 patients (Fig. 2). Enrichment of pCNVs was found in chr7, chr15 and chr22, mainly due to a few common syndromes identified in our cohorts – William Beuren syndrome (29 patients), Prader-Willi/Angelmen syndrome (21 patients), 22q11.2 deletion (5 patients) and 22q13.3 deletion (7 patients), respectively. The chromosomes with less frequent CNVs (frequency below 5) were chr6, chr9, chr14, chr16, chr19, chr20 and chrY. A full list of pCNVs analyzed was included in Additional file 1: Table S1.

### Diagnostic yields in select conditions

When patients were divided to subgroups based on ID severity, the group of severe ID obtained the highest diagnostic yield of 33%, compared with 13–22% for the mild, moderate and profound group (Table 1a). The yield nominally increased with ID severity, though not statistically significant (*p* = 0.084, fisher’s exact, two-tailed).

**Fig. 3:**
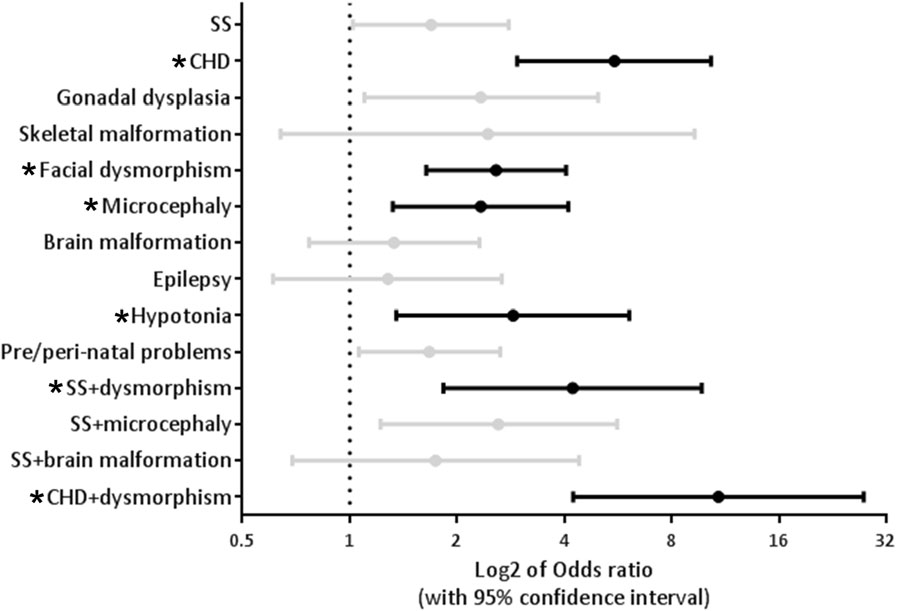
Odds ratio of yielding pCNVs in DD/ID patients with different co-occurring conditions. Log2 of odds ratio were displayed. Odds ratio with a *p*-value< 0.01, two tailed were displayed in black (also a “*” mark before the text), while others were shown in gray. SS:short stature; CHD:congenital heart defects

To assess the diagnostic yields in DD/ID patients with co-occurring conditions, the cohort were divided to subgroups based on the presence of following conditions or their combination: pre/peri-natal problems, family history, short stature, congenital heart defects and twenty other conditions (Table 1, also see Methods for details). The number of patients with pCNVs identified and the diagnostic rate of each group was listed in Table 1 part II/III. Odds ratio (OR) of yielding a pathogenic finding in the presence of select condition was calculated (Table 1.II) and plotted (Fig. 3) when the number of patients with select condition was above 50. Four conditions when co-occurring with DD/ID showed a statistically higher chance of yielding pCNVs - congenital heart defects (55%, OR:5.52), facial dysmorphism (39%, OR:2.57), microcephaly (34%, OR:2.33), hypotonia (35%, OR:2.87) - markedly higher than the 28% overall diagnostic rate. In the presence of two comorbid conditions - short stature and facial dysmorphism, or congeital heart defects and facial dysmorphism, the yield was also markedly elevated (38%, OR:4.22; 62%, OR:10.81, respectively). Karyotypical abnormalities were known in 26 patients prior to CMA. Among these patients, 24 (92%) were identified with pCNVs, including 11 cases with the genomic content of marker chromosomes revealed, 3 cases with gain or loss found in “balanced rearrangement” (based on karyotype), 9 cases with gain/loss confirmed and spanning clarified, and 1 case with pCNV identified in a region other than the structural rearrangement site detected by karyotypical analysis. The rest 2 with no pCNV identified were patients with karyotypic results showing balanced rearrangement.

## Discussion

### More losses than gains in pCNVs

In this study, an increasing proportion of losses were observed with the pathogenic interpretation of CNVs. This is consistent with the notion that many gains present in the human genome are benign. Based on the CNV study on 59,898 exomes by Exome Aggregation Consortium, most phenotypically normal individuals possess higher number of duplications than deletions [8]. The proportion of losses in the pCNVs can also be influenced by the interpretation, as the evidence guiding copy number losses towards pathogenic interpretation is more readily available than gains (in 1247 genes curated by ClinGen expert team, 250 genes have been rated as “sufficient evidence for haploinsufficiency”, while only three genes were rated as “sufficient evidence for triplosensitivity”, https://www.ncbi.nlm.nih.gov/projects/dbvar/clingen/index.shtml).

### The phenotypes associated with pCNV finding

Though CMA has been recommended as the first-tier genetic test for DD/ID, it remains costly and majority of patients could not obtain a diagnosis after the test [9]. Development of next-generation sequencing offers another option for genetic diagnostics of DD/ID. Based on a recent study, whole exome sequencing identified 29.3% conclusive diagnoses in a cohort of 150 patients with complex pediatric neurological conditions [10]. In the foreseeable future, the option between CMA and whole exome sequencing for DD/ID is likely to be put into discussion. Delineating the phenotypic clues of pathogenic CNVs can offer hints for the best cost-effectiveness, though a definite answer should come from the direct comparison of diagnostic yields between next-generation sequencing and CMA.

In our study, diagnostic yield of CMA appeared to correlate positively with ID severity (mild:19%, moderate:22% and severe:33%, Table 1.I), though the correlation was not statistically significant (*p* = 0.08, Fisher’s exact). The number of profound ID cases in our cohort was small to generate a reliable conclusion. A study based on 349 individuals in Italy reported a higher detection rate of causative CNVs in mixed ID (21.5%, IQ < 70) than borderline ID (8.8%, IQ:70–85), but no further categorization of severity in the mixed ID cohort was assessed [11]. Larger datasets are needed to warrant the correlation between ID severity and CMA yield, ideally in a non-syndromic ID cohort.

The comorbidity of congenital heart defects (CHD) in DD/ID is the strongest single phenotype associated with pCNV finding in our study, with an odds ratio of 5.52. When additional comorbid condition is present, the effect size can be even larger - 62% yield was found in DD/ID patients with co-occurring CHD and facial dysmorphism (OR:10.81). This is consistent with a prior study by Shoukier et al., based on CMA results from 342 children with unexplained DD/ID in Europe - they found CHD was more frequently seen in children with pCNVs compared to those with normal array CGH results [12]. There are also reports about higher yields in syndromic CHD with additional phenotypic indications [13, 14]. Geng et al. reported 22.7% detection rate of pathogenic CNVs in CHD patients with co-occurring DD/ID or ASD, compared to 4.3% in isolated cases [15]. Together with our finding, elevated CMA yield was found in comorbidity of CHD and DD/ID, and resorting to CMA is appropriate in such conditions.

Short stature is another comorbid condition frequently seen in our cohort (201/710). The overall yield of DD/ID with short stature was 32%, and increased to 38% when additional feature of dysmorphism or microcephaly was present (Table 1.II). Though the statistical power was less than adequate (*p* = 0.048, Fisher’s exact) in our study, short stature has been reported to be more frequently seen in DD/ID children with pCNVs [12], thus it is a possible indication of pCNV finding. The yield of CMA reported in short stature was between 4% [16] -10% [17], and the difference could be attributed to the varied proportion of syndromic patients.

In cases of DD/ID comorbid with other neurological abnormalities, we found hypotonia (35%, OR:2.87, *p* = 0.006) and microcephaly (34%, OR:2.33, *p* = 0.003) was associated with higher CMA yield, but not epilepsy (24%, *p* = 0.567), brain malformations (24%, *p* = 0.328) or autism (15%) (Table 1, all statistical comparisons were based on fisher’s exact). This is consistent with previous reports - microcephaly and hypotonia, but not epilepsy, were more frequently seen in DD/ID patients harboring pathogenic CNVs than those with normal array results [12, 18]. The documented yield of CMA in patients comorbid of DD/ID and autism varied (12.7% [19], 14% [11], 22% [20] and 26.1% [21]), and in our small subset of 20 patients, three were found to harbor pCNVs, resulting an intermediate yield of 15%.

One of the largest genotype-phenotype analysis so far - Cooper et al. investigated the rare CNV burden on 15,767 individuals in a mixed ID cohorts, and found greater enrichment of CNVs in patients with craniofacial anomalies and heart defects compared to those with epilepsy and autism [22]. Though the burden of rare CNVs cannot directly translate to clinically relevant findings, the overall trend should be informative. Our findings generally agreed that facial dysmorphism and CHD were more indicative of pCNV findings than epilepsy and autism. We additionally found gonadal dysplasia, skeletal malformation and pre/peri-natal problems were not associated with increased yield. The literature on CMA yield in DD/ID comorbid with these conditions is not sufficient, and relevant findings are subjected to further investigation.

A few other conditions, not commonly seen in DD/ID, were reviewed in this study but without statistical comparison performed (Table 1.III). Cleft lip/palate was found in 8 DD/ID patients, and CMA revealed pCNVs in 7 out of 8 patients (88%). These pCNVs were located at different regions, including 7p21 deletion (Saethre–Chotzen syndrome), partial trisomy of chromosome 9, 7q11.23 deletion, 10q26 deletion and 8q22-q24 duplication etc.. Since the reported yield of CMA in cleft lip/palate was between 11 and 14.8% based on larger cohorts [23, 24], our finding of high yield could be incidental as the number of patients was small. Another condition -abnormal biochemical profile - was not associated with elevated diagnostic yield in our DD/ID patients (yield: 27%, versus 29% in patients with normal biochemical results). Recent studies report that inborn errors of metabolism contribute to 1–5% of ID etiology [25]. American Pediatric Association also recommended considering the metabolic screening for children presenting with DD/ID [5]. In our study, patients referred to CMA after metabolic screening were mostly those without clear indication of a monogenic metabolic disorder. Our results did not support the atypical biochemical profile as a phenotypic indication of pCNV finding.

### Karyotypical abnormality referred to CMA

Karyotypical abnormalities were known in some DD/ID patients prior to CMA. They were still referred mainly for further delineating the intervals of genomic aberration. Notably, in the 5 patients with karyotypically balanced rearrangement, 2 were identified to harbor micro-deletion/duplications with clinically relevant CNVs based on CMA. This highlights the possibility of genomic content loss/gain in those karyotypically balanced structural variations.

### Limitations of this study

There are a number of limitations in our study: 1. the sample size is modest, especially those patients with fully accessible clinical information, which limited the statistical power in the comparison of yields based on disease severity and comorbid conditions. 2. Certain conditions can be a matter of clinical judgment like facial dysmorphism, and certain defects may be overlooked in patients without comprehensive evaluation of multiple systems. 3. The diagnostic yield in our study was overall higher than reported, which could be accounted by the patient selection. Patients with karyotypical abnormality were not excluded. In addition, metabolic screening was routinely done in our clinic, which excludes those with identifiable inborn errors of metabolism due to monogenic variants. Nonetheless, the patients in our study, with broad range of phenotypes, can be representative of DD/ID cohorts.

## Conclusions

In conclusion, this study assessed the yield of CMA based on phenotypic features in a highly heterogeneous DD/ID cohort. The results suggest a disparity of gains and losses in identified pCNVs, and varied yields exist in patients with different phenotypic presentation. Congenital heart defects, microcephaly, hypotonia and facial dysmorphism co-occurring with DD/ID associate with an increased probability of pCNV finding. The presence of these comorbid conditions can be among factors to consider when planning CMA on DD/ID patients.

## Additional file

Additional file 1: **Table S1.** The chromosomal location, type and size of pathogenic CNVs reported in this study. (XLSX 31 kb)

## Abbreviations

ACMG: American College of Medical Genetics and Genomics
CHD: Congenital heart defects
CMA: Chromosomal microarray analysis
DD: Developmental delay
DGV: Database of genomic variants
ID: Intellectual disability
LB: Likely benign
LP: Likely pathogenic
pCNVs: Pathogenic copy number variations
VOUS: Variants of uncertain clinical significance
